# Sensitization of Cervical Cancer Cells to Cisplatin by Genistein: The Role of NF**κ**B and Akt/mTOR Signaling Pathways

**DOI:** 10.1155/2012/461562

**Published:** 2012-09-27

**Authors:** K. Sahin, M. Tuzcu, N. Basak, B. Caglayan, U. Kilic, F. Sahin, O. Kucuk

**Affiliations:** ^1^Department of Animal Nutrition, Faculty of Veterinary Medicine, Firat University, 23119 Elazig, Turkey; ^2^Department of Biology, Faculty of Science, Firat University, 23119 Elazig, Turkey; ^3^Department of Genetics and Bioengineering, Faculty of Engineering and Architecture, Yeditepe University, 34755 Istanbul, Turkey; ^4^Department of Physiology, Faculty of Medicine, Yeditepe University, 34755 Istanbul, Turkey; ^5^Department of Medical Biology, Faculty of Medicine, Bezmialem Vakif University, 34093 Istanbul, Turkey; ^6^Winship Cancer Institute, Emory University, Atlanta, GA 30322, USA

## Abstract

Cervical cancer is among the top causes of death from cancer in women. Cisplatin-based chemotherapy has been shown to improve survival; however, cisplatin treatment is associated with toxicity to healthy cells. Genistein has been used as an adjunct to chemotherapy to enhance the activity of chemotherapeutic agents without causing increased toxicity. The present study was designed to investigate the effect of genistein (25 **μ**M) on antitumor activity of cisplatin (250 nM) on HeLa cervical cancer cells. We have examined the alterations in expression of NF-*κ*B, p-mTOR, p-p70S6K1, p-4E-BP1, and p-Akt protein levels in response to treatment. The combination of 25 **μ**M genistein with 250 nM cisplatin resulted in significantly greater growth inhibition (*P* < 0.01). Genistein enhanced the antitumor activity of cisplatin and reduced the expression of NF-*κ*B, p-mTOR, p-p70S6K1, p-4E-BP1, and p-Akt. The results in the present study suggest that genistein could enhance the activity of cisplatin via inhibition of NF-**κ**B and Akt/mTOR pathways. Genistein is a promising nontoxic nutritional agent that may enhance treatment outcome in cervical cancer patients when given concomitantly with cisplatin. Clinical trials of genistein and cisplatin combination are warranted to test this hypothesis.

## 1. Introduction

As of 2008, cervical cancer is the third most common cause of cancer and the fourth most frequent cause of deaths from cancer in women and more than 500,000 new cervical cancer cases and 275,000 deaths were reported worldwide [1]. Although the high incidence rate is disappointing, survival rates of these patients continue to improve with the recent developments in the treatment of this particular cancer type [1, 2]. As the number of studies investigating the application of chemotherapeutical agents as a concomitant treatment method increases, chemoradiotherapy including cisplatin is becoming the recommended method instead of radiotherapy alone [2]. 

Cisplatin (cis-diamminedichloroplatinum II, CDDP), is an effective agent in the treatment of cervical cancer [3]. However, its usage is limited by its toxicity and acquired chemoresistance throughout the course of treatment [4–6]. To this end, targeted therapies that can differentiate between tumor cells and healthy cells are being developed. A naturally occurring soybean isoflavone, genistein, could inhibit tumor growth and induce apoptosis of tumor cells without damaging the normal cells [7–9]. 

Genistein (4′,5,7-trihydroxyisoflavone) has a heterocyclic diphenolic structure that is similar to estrogen, but it has a more potent biological activity [10, 11]. Genistein can inhibit tyrosine kinase and inhibit cancer cell proliferation *in vivo* and *in vitro* without causing toxicity to healthy cells [12]. Studies suggest that genistein can also regulate several signaling pathways in cancer cells and promote cancer cell death. Inhibition of Nuclear Factor-kappa B (NF-*κ*B) and attenuation of Akt pathways by genistein have been shown in various cancer types [13–16]. NF-*κ*B not only controls the expression of genes involved in survival and proliferation, but also plays a key role in apoptosis [17]. Moreover, NF-*κ*B inhibition in tumor cells may result in increased activity of topoisomerase II inhibitors and, hence, this inhibition can be used in anticancer therapy [18].

Phosphatidylinositol 3-kinase (PI3K)/Akt pathway is one of the major growth-factor-induced pathways in tumorigenesis and malignant transformation [19, 20]. Akt pathway activates many downstream signaling pathways responsible for both cell survival and apoptosis [21]. Mammalian target of rapamycin (mTOR) is one of the downstream serine/threonine kinases of PI3K/Akt pathway and regulates cell growth and survival and, thus, it is considered as a valid target for anticancer treatments [22]. mTOR can be either directly phosphorylation-activated by Akt or indirectly activated by Akt through the inhibition of tuberous sclerosis complex 1 and 2 (TSC1/2) and activation of Ras homologue-enriched in brain (Rheb) [23]. mTOR exists as TORC1 and TORC2 complexes. In TORC1 complex, it initiates translation by eukaryotic translation initiation factor (eIF4E) binding proteins (4EBP1) and by ribosomal p70S6 kinase (p70S6K). When mTOR protein phosphorylates 4E-BP1, it dissociates from eIF4E. Once eIF4E is freed from 4e-BP1, it can form complex structures with several other proteins, including eIF4G or eIF4F. When mTOR phosphorylates p70S6K, this kinase phosphorylates S6 ribosomal protein in return [24, 25]. S6 kinase can catalyze phosphorylation and inhibition of insulin receptor substrate (IRS) proteins; then IRS proteins can no longer activate PI3K pathway and this results in an indirect inhibitory effect on Akt [26, 27]. mTOR can also phosphorylate Akt through a possible positive feedback mechanism [28]. 

In this study, we hypothesized that cisplatin treatment administered together with genistein could potentiate cervical cancer growth inhibition *in vitro* through downregulation of mTOR pathway. To test our hypothesis, we evaluated the effects of genistein and cisplatin on cell growth and apoptosis-related gene expression in HeLa human cervical cancer cell line.

## 2. Materials and Methods

### 2.1. Cell Culture and Reagents

The human cervical cancer cell line, HeLa cells (American Type Culture Collection, Manassas, VA) was maintained in RPMI-1640 medium containing 10% heat inactivated fetal bovine serum, 1% L-glutamine, 100 U/mL penicillin G, and 100 *μ*g/mL streptomycin. Cells were incubated in a humidified, 5% CO_2_ atmosphere at 37°C. No growth factors were added to the cell culture medium at any time. Genistein (Sigma Chemical Co., St. Louis, MO, USA) was dissolved in 0.1 M Na_2_CO_3_ to make a 10-mM stock solution. Cisplatin (Sigma Chemical Company, St. Louis, MO) was dissolved in phosphate buffered saline (PBS) to make a 0.5 mM stock solution.

### 2.2. Cell Viability Assay

Cell viability was determined by MTS Assays. HeLa cells were seeded 3000 cells in a 96-well plate and incubated overnight. Cells (2–5 × 10^4^) were treated with genistein (25 *μ*M), cisplatin (250 nM), and their combination treatment for 24 hours. After 24 hours of total treatment, the cells were incubated at 37°C with 1 mg/mL MTT reagent (Sigma, St. Louis, MO) for 2 hours. The formazan crystals were dissolved in isopropanol. Spectrophotometric absorbance of the samples was determined by the ULTRA Multifunctional Microplate Reader (ELx800-BIO-TEK) at 490 nm. 

### 2.3. Western Blot Analysis

HeLa cells were treated with genistein (25 *μ*M), cisplatin (250 nM), and the combination treatment for 24 hours. The total proteins from these samples were extracted. These total proteins were resolved through sodium dodecyl sulfate polyacrylamide gels and then were transferred to a nitrocellulose membrane. After blocking with 5% nonfat dry milk, the membrane was incubated with anti-NF-*κ*B p65, anti-mTOR, anti-70S6K1, anti-4E-BP1, and anti-Akt (Abcam, Cambridge, UK). Primary antibody was diluted (1 : 1000) in the same buffer containing 0.05% Tween-20. The nitrocellulose membrane was incubated overnight at 4°C with protein antibody. The blots were washed and incubated with horseradish peroxidase-conjugated goat anti-mouse IgG (Abcam, Cambridge, UK). Specific binding was detected using diaminobenzidine and H_2_O_2_ as substrates. Protein loading was controlled using a monoclonal mouse antibody against *β*-actin antibody (A5316; Sigma). Blots were performed at least three times to confirm data reproducibility. Bands were analyzed densitometrically using an image analysis system (Image J; National Institute of Health, Bethesda, USA). 

### 2.4. Statistical Analysis

To determine the difference in cell viability between experimental sets of cervical cancer cell line, experiments were repeated at least three times and SPSS was used for statistical analysis. Comparisons of treatment outcome were tested for statistical difference by the paired *t*-test. Statistical significance was assumed at a *P* value of <0.05.

## 3. Results

### 3.1. Genistein Enhances the Inhibitory Effect of Cisplatin on the Proliferation of HeLa Cells

The effects of genistein, cisplatin, and their combination on the proliferation of HeLa cells were evaluated with MTS assay. In MTS assay, cells are treated with a tetrazolium compound, MTS (3-[4,5-dimethylthiazol-2-yl]-2,5-diphenyltetrazolium bromide). Since metabolically active cells can reduce MTS to insoluble purple formazan dye products, relative cell viability for each treatment compared to control was measured [29]. Data from this assay showed that combination treatment of genistein and cisplatin enhances the inhibition of cellular growth in HeLa cells. 

**Figure 1:**
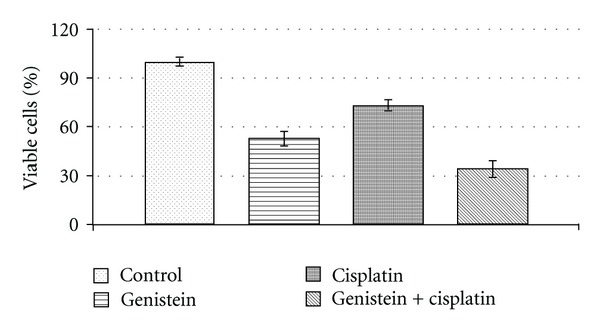
Growth inhibition of human cervical cancer cell lines HeLa treated with genistein, cisplatin, and the combination treatments were evaluated by the MTT assay. HeLa cells were treated with genistein (25 *μ*M), cisplatin (250 nM), and the combination treatment. *P < 0.05; **P < 0.01.

The rationale for choosing 250 nM cisplatin and 25 *μ*M genistein came from our previous observation that revealed a marked inhibition of cell growth in human cancer cells [7]. HeLa cells were treated with cisplatin and genistein alone and in combination of the two for 24 hours. When compared to controls, combination treatment inhibited the proliferation of HeLa cells to significantly higher extend than either treatment alone. Percent of viable cells after the combination treatment decreased to ~30%, while percents of viable cells for genistein treatment alone and for cisplatin treatment alone were ~50% and ~70%, respectively (Figure 1). These results suggest that combination of genistein with cisplatin elicited significantly greater growth inhibition in HeLa cells compared to either agent alone. Since we found that genistein could potentiate the inhibition of cancer cell growth, we next tested the expression of possible target proteins which may be involved in the mechanism of genistein and cisplatin.

### 3.2. Genistein Prevents Cisplatin-Induced Upregulation of NF-*κ*B in HeLa Cells

**Figure 2:**
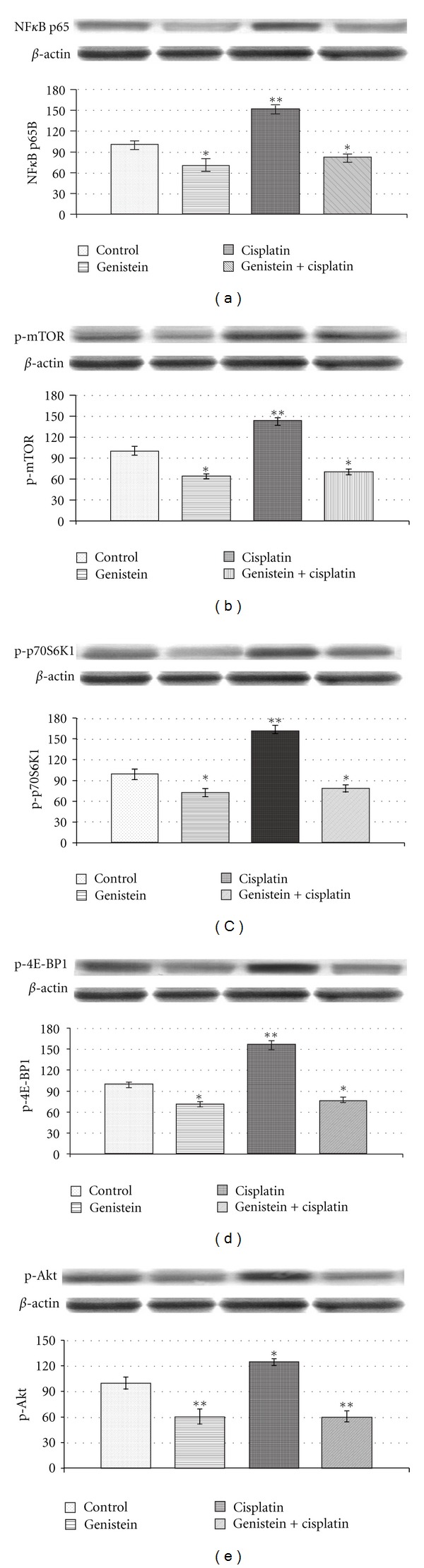
The intensity of the bands was quantified by the densitometric analysis. The expression of (a) NF-*κ*B, (b) p-mTOR, (c) p-p70S6K1, (d) p-4E-BP1, and (e) p-Akt in HeLa cells. Cells untreated or treated with 25 *μ*M genistein, 250 nM cisplatin (Cis), and the combination (genistein + cisplatin). *β*-actin antibodies were used as internal controls for equal loading of proteins. Data are percent of the control. *P < 0.05; **P < 0.01.

NF-*κ*B is a transcription factor which plays an important role in apoptosis mechanisms by exerting its regulatory effects on survival genes. Expression level of NF-*κ*B was evaluated at the protein level. By Western Blot analysis, we examined the expression level of NF-*κ*B p65B, a subunit of NF-*κ*B transcription complex, in genistein alone, cisplatin alone, and genistein-plus-cisplatin-treated HeLa cells. Cisplatin alone increased the expression level of NF-*κ*B p65B up to 150%, compared to control, whereas genistein downregulated this subunit to ~75%. When genistein is added to cisplatin treatment, expression level of NF-*κ*B p65B protein is reduced to ~80%. These results suggest that genistein can downregulate the increased expression level of NF-*κ*B induced by cisplatin in HeLa cells (Figure 2(a)). 

### 3.3. Genistein Inhibits Cisplatin-Induced Activation of mTOR Pathway in HeLa Cells

In order to evaluate the involvement of mTOR molecular pathway in the antiproliferative effect of cisplatin and genistein, we assessed the expression levels of p-mTOR, p-p70S6K1, p-4E-BP1, and p-Akt in HeLa cells treated with genistein and/or cisplatin. Genistein reduced the level of phosphorylated mTOR, p70S6K1, 4e-BP1, and Akt induced by cisplatin in HeLa cells (Figures 2(b), 2(c), 2(d), and 2(e)). mTOR is known to regulate initiation of translation through two pathways: S6K and 4E-BP1. As a decrease in expression of mTOR would cause a decrease in expression of these two molecules, this hypothesis is supported by our data. 

## 4. Discussion

There is a need to develop more efficient treatment strategies to increase the efficacy of existing therapies while not compromising the normal cells. Cisplatin is one of the most effective anticancer agents in the treatment of cervical cancer; however, it is associated with severe toxicity and acquired drug resistance after therapy. Severe renal, neurologic, and gastrointestinal side effects and acquired chemoresistance are the major reasons of cisplatin treatment failure. To overcome the limitations of cisplatin treatment, combination with targeted therapy using naturally occurring compounds was suggested [30–32]. Many natural compounds with known anticancer activity have been used including sulforaphane [33, 34] and genistein [35]. In the light of these previous studies we chose genistein as a nontoxic nutritional agent to augment the efficacy of cisplatin treatment in HeLa cells.

In this study, we observed the superiority of genistein plus cisplatin combination compared to cisplatin alone in inhibition of the growth of HeLa cervical cancer cells *in vitro*. The effect of genistein on cisplatin's anticancer activity has been previously reported for ovarian cancer [36] and pancreatic cancer [35]. We investigated the therapeutic effect of genistein and cisplatin in the HeLa cells and found a statistically significant inhibition of cell growth when cells were treated with a combination of genistein and cisplatin, compared to either agent alone. Growth inhibition of HeLa by cisplatin was augmented by genistein, thereby obviating the need to further increase the concentration of cisplatin. The effect of genistein-mediated enhanced efficacy of cisplatin in cervical cancer cells was demonstrated for the first time in this study.

Activation of mTOR signaling pathway is associated with cell survival in cervical cancer cells [37]. We have found increased mTOR expression after cisplatin treatment which could be prevented by the addition of genistein, a mechanism first shown in the present study. The combination of cisplatin and genistein could be a promising strategy in the treatment of cervical cancer. mTOR activity can be monitored by phosphorylation of S6K, 4E-PB1 proteins [38]; we also observed increased expression of these proteins with cisplatin treatment which could be abrogated by genistein. When HeLa cells were treated with cisplatin alone, the expression levels of phosphorylated mTOR, p70S6K1, and 4E-BP1 increased up to 140%, 170%, and 150%, respectively. However, addition of genistein downregulated these cisplatin-induced proteins by 70%. These results suggest that cisplatin upregulates mTOR pathway and genistein prevents this upregulation by downregulating phosphorylated p70S6K1 and 4E-BP1 proteins. We also observed that this activity is associated with downregulation of phosphorylated Akt, which suggests that decrease in the expression of mTOR pathway is probably mediated via Akt or a decrease in this pathway negatively regulates and inactivates Akt.

In this study, we also investigated the effects of genistein and cisplatin on NF-*κ*B, which is known to be upregulated upon cisplatin treatment [36]. Similar to previous observations, genistein decreased the expression of cisplatin-induced NF-*κ*B in HeLa cells. These results suggest a molecular mechanism involving both NF-*κ*B and mTOR pathways induced by cisplatin and inhibited by genistein.

## 5. Conclusion

In conclusion, cisplatin treatment is potentiated with genistein in HeLa cells by regulating NF-*κ*B, Akt, and mTOR pathways which are critical for cell survival and apoptosis. Our findings suggest that cisplatin and genistein combination could be used to improve the treatment outcome in cervical cancer. This combination is a less toxic option in the treatment of cervical cancer, especially in the presence of chemoresistance to cisplatin. Future clinical trials are warranted to investigate the combination of cisplatin and genistein in patients with cervical cancer.
